# 2023 European Society of Cardiology guidelines for the management of infective endocarditis: Statement of endorsement by the NVVC *Full version*

**DOI:** 10.1007/s12471-025-02010-w

**Published:** 2025-12-18

**Authors:** Annelot J. L. Peijster, Cees van Nieuwkoop, Ruud W. M. Keunen, Susanne Felix, Berend J. van Welzen, Ilse J. E. Kouijzer, C. H. Edwin Boel, Nelianne J. Verkaik, Ka Yan Lam, Robert J. M. Klautz, Andor W. J. M. Glaudemans, Ricardo P. J. Budde, Alexander H. Maass, Reinoud E. Knops, Otto Kamp, Wilco Tanis

**Affiliations:** 1 Department of Cardiology, Amsterdam University Medical Centre, Amsterdam, The Netherlands; 2 Department of Cardiology, Haga Teaching Hospital, The Hague, The Netherlands; 3 Department of Internal Medicine, Haga Teaching Hospital, The Hague, The Netherlands; 4 Department of Neurology, Haga Teaching Hospital, The Hague, The Netherlands; 5 Department of Cardiology, Catharina Hospital, Eindhoven, The Netherlands; 6 Department of Infectious Diseases, University Medical Centre Utrecht, Utrecht, The Netherlands; 7 Department of Internal Medicine, Radboud University Medical Centre, Nijmegen, The Netherlands; 8 Department of Medical Microbiology, University Medical Centre Utrecht, Utrecht, The Netherlands; 9 Department of Microbiology, Erasmus Medical Centre, Rotterdam, The Netherlands; 10 Department of Cardiothoracic Surgery, Catharina Hospital, Eindhoven, The Netherlands; 11 Department of Cardiothoracic Surgery, Amsterdam University Medical Centre, Amsterdam, The Netherlands; 12 Department of Cardiothoracic Surgery, Leiden University Medical Centre, Leiden, The Netherlands; 13 Department of Nuclear Medicine and Molecular Imaging, University Medical Centre Groningen, Groningen, The Netherlands; 14 Department of Radiology & Nuclear Medicine, Erasmus Medical Centre, Cardiovascular Institute, Rotterdam, The Netherlands; 15 Department of Cardiology, University of Groningen, University Medical Centre Groningen, Groningen, The Netherlands

**Keywords:** Infective endocarditis, Infection, Endocarditis Team, Prevention, Cardiac imaging, Diagnostic criteria, Microbiological diagnosis, Prosthetic valve endocarditis, Device endocarditis, Cardiac surgery

## Abstract

This review evaluates the 2023 European Society of Cardiology (ESC) guidelines for the management of infective endocarditis (IE) and offers insights into topics particularly relevant to clinical practice in the Netherlands. The multidisciplinary IE Working Group assessed all ESC recommendations and concluded that the majority could be endorsed, albeit with certain modifications. The IE Working Group presents a refined (and simplified) antibiotic prophylactic regimen and an updated table to guide the analysis of suspected blood culture-negative IE. Furthermore, the pivotal role of the Endocarditis Team in the management of patients with suspected and confirmed IE was reaffirmed, along with reinforced recommendations for nearly all cardiac and extracardiac imaging in these cases. Notably, a preliminary recommendation was issued for switching to oral antibiotic therapy in patients with native valve endocarditis caused by viridans streptococci, while awaiting the revision of the Dutch Working Group on Antibiotic Policy (SWAB) IE guideline. In addition, the surgical recommendations were evaluated and revised, including improved clinical criteria in case of cardiac surgery following neurological complications of IE and an advised disregard of the new class I ESC recommendation for surgery in early prosthetic valve endocarditis (< 6 months). Moreover, an additional device recommendation was proposed for the choice of (alternate) devices in case of device reimplantation after IE.

**Supplementary Information:**

The online version of this article (10.1007/s12471-025-02010-w) contains supplementary material, which is available to authorized users.

## Introduction

In October 2023, a new European Society of Cardiology (ESC) guideline for the management of infective endocarditis (IE) was released, updating the 2015 guideline [1, 2]. A multidisciplinary IE Working Group was initiated by the Dutch Society of Cardiology (Nederlandse Vereniging voor Cardiologie, NVVC) to review how the 2023 ESC guidelines for IE management could be effectively implemented in the Netherlands. A key distinction of the IE Working Group, compared to the ESC Task Force, is the inclusion of clinical microbiologists and a neurologist, along with an overall balanced representation of various medical specialists. The new guideline recommendations focus on prevention, diagnostic criteria, multimodality imaging, oral antibiotic therapy, surgical indications, and timing [1]. Besides these main themes, this guideline also reinforced their recommendation of the Endocarditis Team (with an upgrade from class IIa, level B to class I, level B) and highlights patient-centered care and shared decision-making in IE. The IE Working Group supports these recommendations as the Endocarditis Team enhances evidence-based practice by integrating guideline and evidence discussions, individual (clinical) patient information, and the experience and expertise of all involved specialists. And while the emphasis on patient-centered care and shared-decision making is always important in healthcare, the IE Working Group confirms that the heterogeneity, severity, complexity, and prolonged treatment duration of IE should underscore their significance. Before further evaluating the guideline, it should be noted that most recommendations of this specific ESC guideline rely on low-level evidence; with 56% of the recommendations based on level C evidence and only 3% supported by level A evidence [1, 3]. This paper summarises the recommendations of the IE Working Group, proposing several adjustments to the ESC guideline, at least within the Dutch context.

## Prevention and prophylaxis

The new guideline introduced many new and revised recommendations in IE prevention. These include recommendations on general prevention measures and on antibiotic prophylaxis before (predominantly oro-dental) procedures.

The IE Working Group endorses all general preventive measures. In the ESC guideline, a new distinction was made between intermediate risk and high-risk patients, of which the intermediate risk group consists of patients with rheumatic heart disease, non-rheumatic degenerative valve disease, congenital valve abnormalities including bicuspid aortic valve disease, cardiovascular implanted electronic devices (CIED) and hypertrophic cardiomyopathy. High-risk patients include patients with previous IE, prosthetic valves, ventricular assist devices and congenital heart disease (not including isolated congenital valve abnormalities) such as untreated cyanotic congenital heart disease or those treated with post-palliative shunts, conduits, or other prostheses. For high-risk patients, it is recommended to follow all preventive and prophylactic measures, while the intermediate risk group is only advised to follow general prevention measures. The new ESC guideline also includes a new IE education card for high-risk patients to help prevent IE [1]. Certainly, patient education and awareness are strongly recommended by the IE Working Group. Therefore, along with this endorsement paper, a patient IE card (including QR-code to facilitate sharing and digital storing) will be introduced and distributed for use in the Netherlands (Fig S1 in the Supplementary Appendix).

**Fig. 1:**
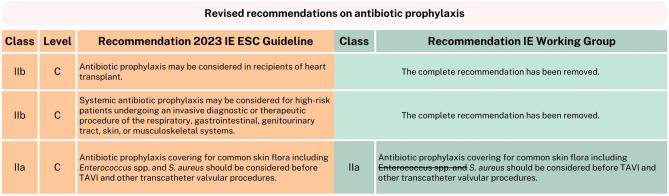
Revised recommendations on antibiotic prophylaxis. *IE* infective endocarditis, *ESC* European Society of Cardiology, *S.* *aureus* Staphylococcus aureus, *TAVI* transcatheter aortic valve implantation

As for the recommendations for antibiotic prophylaxis, the IE Working Group supports nearly all, with the exception of two new recommendations and proposes to adjust another (Fig. 1). The first is the class IIb, level C recommendation for antibiotic prophylaxis in recipients of heart transplant undergoing oro-dental procedures. We found insufficient evidence to support this new recommendation, as the cited studies and review include only a small number of IE heart transplantation patients. Within this limited group, they not only highlight the variety of causative organisms (including frequent fungi) for which the proposed prophylaxis would often be inadequate, they also raise uncertainty whether the time of origin is peri-procedural (with a median onset of IE at 8.4 (IQR 3.0, 35.8) months after transplantation) [4–6]. Moreover, we advise adhering to the local heart transplantation guideline on this matter, as current evidence does not justify a change in practice [7]. Another new IIb, level C recommendation is to consider systemic antibiotic prophylaxis for high-risk patients undergoing an invasive diagnostic or therapeutic procedure of the respiratory, gastrointestinal, genitourinary tract, skin, or musculoskeletal systems [8, 9]. We conclude that more evidence is needed before making such formal new recommendation and encourage following the Dutch Working Group on Antibiotic Policy (Stichting Werkgroep AntibioticaBeleid, SWAB) guidelines for antibiotic recommendations for high-risk procedures associated with bacteraemia. Finally, an adjustment was made by the IE Working Group to the IIa, level C recommendation for antibiotic prophylaxis before transcatheter aortic valve implantation (TAVI) and other transcatheter valvular procedures. The advice is to cover for common skin flora, including *S. aureus* (*Staphylococcus aureus*) and now *Enterococcus *spp. as well [1]. While *Enterococcus* spp. are a frequent cause of TAVI endocarditis, it is uncertain whether the occurrence of enterococcal TAVI endocarditis is related to the procedure itself, and therefore, whether prophylaxis before TAVI procedures should be adjusted [10, 11]. The definition of ‘early’ IE varies significantly across the performed studies (ranging from 2 months to 1 year) and in most studies, the majority of cases have a potential source of entry identified (other than the valve implantation) [12, 13]. In addition, similar numbers were observed in endocarditis of surgical aortic valve prosthesis caused by enterococci (for which no *Enterococcus* spp. prophylaxis is advised either), while the TAVI population would be presumed to be at greater risk for IE [14, 15]. Therefore, in the absence of concrete evidence, the recommendation has been revised to cover *S. aureus* exclusively. The IE Working Group does emphasize the need to perform research on this matter.

In addition, the IE Working Group proposes a new prophylactic antibiotic regimen for high-risk dental procedures (Tab. 1). In the new ESC guideline, clindamycin was removed as an option due to studies indicating adverse drug reactions, primarily associated with *Clostridioides difficile* infections [16, 17]. Since only one of these studies evaluated clindamycin as a prophylactic agent, we do recommend its continued use in cases of (confirmed) penicillin allergy. In addition, the IE Working Group strives to simplify the recommended regimen, resulting in the removal of antibiotic options for which EUCAST MIC breakpoints are lacking (such as doxycycline and azithromycin) or where there is a risk of potential cross-reactivity (such as cephalexin) [18–20]. Furthermore, the paediatric dosages included in the table of the new guideline were not adopted, as they do not fall within the scope of this guideline (as paediatric IE was also not addressed elsewhere in the guideline).

**Table 1:** Revised prophylactic antibiotic regime for high-risk dental procedures.

Situation	Medication	
	First choice	Alternative
No penicillin/ampicillin allergy	Amoxicillin 2 g, oral	Ceftriaxon 1 g, i.v.
Penicillin/ampicillin allergy	Clindamycin 600 mg, oral	Ceftriaxon 1 gr, i.v.

*G* gram, *i.v.* intravenous, *mg* milligram.

## Imaging

In IE multimodality imaging, the use of cardiac computed tomography (CT), positron emission tomography and computed tomography (PET/CT), as well as cerebral magnetic resonance imaging (MRI), has led to major improvements in diagnostics (especially of prosthetic valve endocarditis (PVE)) [1, 21–23]. The new guideline reinforces and further upgrades most indications for all cardiac and extracardiac imaging for (suspected) IE patients. While the IE Working Group endorses the majority of the new imaging recommendations, it remains crucial (and may be regarded as self-evident) to employ these imaging techniques only when the results offer clinically relevant information and/or a potential change in the management of IE.

**Fig. 2:**
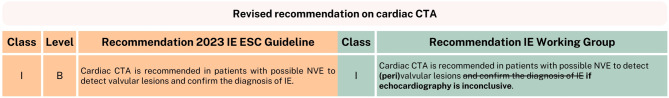
Revised recommendations on cardiac CTA. *CTA* computed tomography angiography, *IE* infective endocarditis, *ESC* European Society of Cardiology, *NVE* native valve endocarditis

The guideline updated and upgraded its recommendation on transoesophageal echocardiogram (TOE) to a class I, level C indication for TOE in patients with suspected IE, even in cases with positive transthoracic echocardiogram (TTE), except in isolated right-sided native valve IE with good quality TTE examination and unequivocal echocardiographic findings. The IE Working Group endorses this recommendation, as it will stimulate consideration of a TOE more quickly, and in all IE-related cases, however, we also emphasize that no new evidence supports this upgrade. Given that it is a semi-invasive procedure, the clinical implications should be carefully considered, particularly in cases that are ineligible for surgery and require antibiotic treatment similar to that for IE, regardless of the TOE [24]. The new class I, level B recommendation for ‘cardiac computed tomography angiography (CTA) in patients with possible native valve endocarditis (NVE) to detect valvular lesions and confirm the diagnosis of IE’ is changed by the IE Working Group by stating (peri)valvular instead of valvular, adding ‘if echocardiography is inconclusive’ and deleting ‘confirm the diagnosis of IE’ (Fig. 2; [25]). Although CTA may offer somewhat limited additional value in this patient group with possible NVE, we agree it can be useful to have the option to perform a CTA when suspicion of IE persists (and echocardiography yielded negative or inconclusive results). It should be noted that performing a CTA in patients with possible NVE may lead to a negative result, which does not rule out endocarditis, as vegetations may go undetected on CTA.

## Diagnostic criteria

In addition to the new ESC guideline featuring updated major and minor criteria for the diagnosis of IE, the revised modified Duke criteria, the Duke-International Society for Cardiovascular Infectious Disease criteria (Duke-ISCVID), were also published last year [26]. Both new criteria have shown to increase sensitivity compared to the 2000 modified Duke criteria and the 2015 ESC criteria; however, the Duke-ISCVID appears to preserve specificity better than the 2023 ESC criteria [27–29]. According to both these diagnostic tools, the diagnosis of IE is considered definite when two major criteria or one major and three minor criteria are met. In rare cases, five minor criteria without any major criteria may also be regarded as definite IE.

**Fig. 3:**
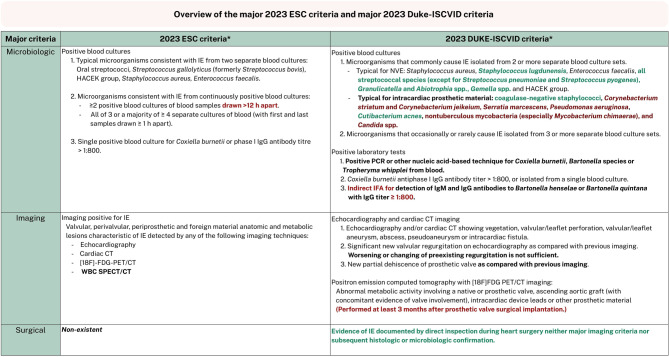
Overview of the major 2023 ESC criteria and major 2023 Duke-ISCVID criteria [1, 26]. *ESC* European Society of Cardiology, *Duke-ISCVID* Duke—International Society for Cardiovascular Infectious Diseases Criteria, *HACEK group* Haemophilus parainfluenzae, Haemophilus aphrophilus, Aggregatibacter actinomycetemcomitans, Cardiobacterium hominis, Eikenella corrodens, and Kingella kingae, *h* hour, *NVE* native valve endocarditis, *PCR* polymerase chain reaction, *IgM* Immunoglobulin M, *IgG* Immunoglobulin G, *CT* computed tomography, *[18F]-FDG-PET/CT* 18F-Fluorodeoxyglucose positron-emission tomography/computed tomography, *WBC SPECT/CT* white blood cells single photon emission computed tomography/computed tomography. *Please note, this table provides a concise overview and not the full published text. The main differences between the two criteria are highlighted in bold, while agreement or disagreement by the IE Working Group is indicated in green and red, respectively, as detailed in the text

The IE Working Group has no comments on the minor ESC criteria. However, it generally favors the major criteria of the Duke-ISCVID criteria, being more applicable in practice (e.g., less stringent timing for blood culture collection [30] and the inclusion of causative pathogens specific to PVE). The Duke-ISCVID criteria comprise three major criteria, compared to two in the new ESC criteria (Fig. 3).

First, the microbiologic major criteria include a more extensive list of causative pathogens marked as typical, especially with the addition of typical bacteria for intracardiac prosthetic material. While the IE Working Group welcomes this change (acknowledging the prevalence of PVE), we recommend limiting it to typical agents for infection of prosthetic valves as opposed to all intracardiac prosthetic material (e.g., left ventricular assist devices) and therefore retaining only *Cutibacterium acnes* and coagulase-negative staphylococci as typical causative pathogens [31]. The other pathogens are less associated with IE, which also aligns with the current standard antibiotic treatment for PVE [1, 26, 32]. Moreover, we propose to revise the microbiologic criterion for ‘indirect immunofluorescence assays (IFA) for detection of IgM and IgG antibodies to *Bartonella henselae* or *Bartonella quintana* with immunoglobulin G (IgG) titer ≥ 1:800’ to retain a ‘high antibody titer to *Bartonella henselae* or *Bartonella quintana’ *solely’, since IFA is not always available. The preferred method may vary between microbiologic laboratories; in the Netherlands, Enzyme Linked Immune Sorbent Assay (ELISA) or Indirect chemiluminescent immunoassay (CLIA) are more reflective of standard practice. Since the cut-off is not well defined for tests other than IFA, a Bartonella PCR should be performed in addition to serology in case of blood-culture negative IE (Fig. 3; Tab. 2).

**Table 2:** Table to follow in case of negative blood cultures for three or more days and a high suspicion of IE.

In case of appropriate negative blood cultures without antimicrobial therapy for three or more days and a high suspicion of IE, do the following*:
Always consult a medical microbiologist, an internist-infectious disease specialist and discuss the patient in the Endocarditis Team
Switch to antibiotic therapy for blood culture negative IE
Consider extending incubation time up to 14 days**
Do serology for *Bartonella *spp. (IgM, IgG) and *Coxiella burnetii*
On indication: do *Brucella *spp. serology and/or mycobacterial blood cultures
Specific PCRs on blood (and consider 16S PCR): *Tropheryma whipplei, Bartonella* spp., *Coxiella burnetii*
16S PCR, specific PCRs *Bartonella* spp., *Coxiella burnetii, Tropheryma whipplei* and consider 18S/ITS PCR on surgical material
Consider non-infectious causes of endocarditis (e.g. systemic lupus erythematosus, neoplasms)

*IE* infective endocarditis, *PCR* polymerase chain reaction.

*If the diagnostic test is not available, send the blood samples and/or blood cultures to a reference laboratory.

**So as not to miss *Cutibacterium acnes* and/or if blood cultures were drawn while receiving antimicrobial therapy.

Second, while we suggest that the imaging major criteria from the new ESC guideline may be utilized, it is important to clearly delineate the differences. The ESC imaging criteria has become open to broad interpretation as it states to be present with any ‘valvular, perivalvular/periprosthetic and foreign material anatomic and metabolic lesions characteristic of IE detected by echocardiography, cardiac computed tomography, 18F-Fluorodeoxyglucose positron-emission tomography/computed tomography (FDG-PET/CT) or white blood cell single photon emission computed tomography (WBC SPECT/CT)’. This differs from both the 2015 ESC guideline and the Duke-ISCVID criteria, which are more specific in naming the potential findings, such as vegetation or abscess. As it is less precise, it also omits the note that FDG-PET/CT should only be performed at least 3 months after prosthetic valve implantation to provide a major criterion (while within 3 months could result in a minor criterion) [1, 22, 26]. The IE Working Group agrees that the 3‑month timing of FDG-PET/CT can be disregarded, but for appropriate interpretation, the timing of the FDG-PET/CT after prosthetic valve implantation should be considered. Although this issue remains debated, we believe it is important to take into account the timing in combination with the surgical technique, the surgical materials used, and the uptake pattern of the FDG-PET/CT, following multiple studies including the TEPvENDO study as well as a Dutch multicenter study [33–36]. Moreover, consultation with an experienced specialist is strongly recommended for proper assessment of the FDG-PET/CT scan, particularly in this population. Besides this remark, the IE Working Group does agree with the new, broader imaging criteria of the ESC guideline, while stressing to be more aware of the fact that the assessment now is increasingly dependent on the individual(s) interpreting the results. Third, the new major criterion, exclusive to the Duke-ISCVID criteria, is the surgical criterion, which indicates being positive when there is documented evidence of IE observed during inspection in cardiothoracic surgery, without the need for confirmation of imaging, histology or microbiology. The IE Working Group believes that the surgeon’s observations should constitute as a major criterion, especially since the patient then has a new prosthetic valve in (or near) the area where signs of IE were observed.

## Blood culture-negative endocarditis

In the Netherlands, the current recommendations on the analysis of (possible) blood culture-negative IE differ from the ESC guideline [1, 37]. The IE Working Group recommends adhering to the Dutch guideline Addendum Infective Endocarditis of the NVVC on this matter, however, it suggests further simplifying them to align with current (Dutch) practice (Tab. 2; [37]). As opposed to the previously recommended diagram by the NVVC [37], serology and polymerase chain reaction (PCR) on blood for *Legionella* spp. and *Mycoplasma* spp. were now removed, as these are extremely rare causes of IE and routinely performing diagnostics may introduce unnecessary uncertainties and costs (as false-positive IgG results for *Mycoplasma* spp. may occur) [38, 39]. In accordance with this, the advice to inoculate blood culture bottles after incubation on agar plates was also removed. Furthermore, *Brucella *spp. as a cause of IE is also quite rare; however, performing serology remains recommended when indicated. In addition, the table advises consultation with an internist-infectious diseases specialist or clinical microbiologist in case of the proposed analyses on indication and states to possibly consider consultation with the Endocarditis Team [37]. The IE Working Group recommends consulting these specialists more promptly whenever blood culture-negative IE is suspected, consistent with our endorsement of the Endocarditis Team. Finally, in line with Dutch practice, 18S/ITS PCR for the detection of fungal infections is now only recommended for consideration.

## Antimicrobial therapy

**Fig. 4:**
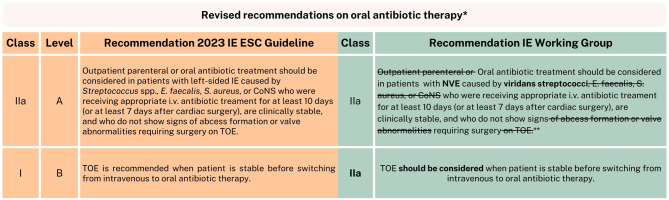
Revised recommendations on oral antibiotic therapy. *IE* infective endocarditis, *ESC* European Society of Cardiology, *E.* *faecalis* Enterococcus faecalis*,* *S.* *aureus* Staphylococcus aureus, CoNS, coagulase-negative staphylococci, *i.v.* intravenous, *TOE* transoesophageal echocardiogram, * Please note, these revised recommendations are preliminary, in anticipation of the new IE Dutch Working Group on Antibiotic Policy (Stichting Werkgroep Antibiotica Beleid, SWAB) guideline. ** The by IE Working Group recommended oral antibiotic therapy for this population (NVE caused by *viridans* streptococci) is amoxicillin 1000 milligrams 4 times/day (with a minimum inhibitory concentration for penicillin of < 0.25 μg/mL)

Generally, for the antimicrobial therapy of IE, the recommendation is to adhere to the SWAB guidelines instead of the ESC guideline, as these are adapted to fit the Dutch context [32]. These guidelines are meticulously developed by multidisciplinary expert groups in the Netherlands and are based on local resistance patterns, while also considering practical factors such as availability and experience with specific antibiotics. However, the IE Working Group would like to address the new class IIa, level A recommendation for outpatient oral antibiotic treatment following the POET (randomized controlled) trial [40]. In light of this, we aim to offer a cautious and provisional recommendation for oral therapy in uncomplicated NVE caused by viridans streptococci, while awaiting the revision of the IE SWAB guideline, which is currently under development (with coinciding specialists from this IE Working Group) (Fig. 4).

Alongside the 5‑year follow-up of the POET trial, the Danish POETry study was published as well, confirming the feasibility of partial oral treatment for a sizable group of IE patients (240 patients) in real-world clinical settings [40, 41]. We recognize the potential for oral therapy and are eager to implement this in the Netherlands. However, there remain several underrepresented and high-risk subgroups, for which we believe it is too early to make definitive recommendations based on the current data. For example, around 30% of patients had PVE in the POETry study, and they were included equally in both treatment groups. However, the causative pathogens of PVE patients were not stated. In the POET trial, they were stated, and only 7 patients with *S. aureus* PVE received partial oral treatment, and as this was a randomized trial, it suggests there were even less *S. aureus* PVE patients in the partial oral group in the POETry study (with its observational design). Therefore, the IE Working Group believes caution is still warranted in all staphylococcal, enterococcal, and PVE infections. We are still awaiting trial results, such as the SNAP trial, and it is also important to considerthe higher risk in certain subgroups, such as enterococcal endocarditis, which is more often associated with relapse infections (and intravascular materials) [40, 42–44]. This predominantly leaves patients with NVE caused by viridans streptococci, for whom we do recommend considering partial oral treatment: amoxicillin 1000 milligrams orally 4 times/day (in case of a minimum inhibitory concentration [MIC] for penicillin of < 0.25 μg/mL) [40, 44–49]. This contradicts the dual therapy approach in the POET trial and POETry study. However, we could not find the rationale to add another antibiotic (such as rifampin) in combination with the amoxicillin for this specific patient group. Moreover, the IE Working Group specifically advises this for switching to oral therapy, excluding the outpatient parenteral option, which is part of the recommendation of the ESC guideline. For cases eligible for outpatient parenteral antimicrobial therapy, we recommend following the SWAB practical guide [50].

The optimal timing for transitioning from intravenous to oral therapy remains uncertain. According to the ESC guideline, a switch is recommended after 10 days (or 7 days in case of surgery), once clinical and biochemical criteria are met and provided there are no contraindications. These include concerns regarding oral therapy absorption in the gastrointestinal tract or issues with treatment adherence [1]. Studies indicate a lack of consensus on duration, and the median time in the POET trial also exceeded their recommendation of 7 days [40, 44, 47, 48]. In addition, this heterogenous population likely requires varying durations before the optimal moment to switch. However, the IE Working Group for now agrees with the recommended number of days (provided all other criteria are met), as it offers useful guidance for the treating clinician. We have one additional comment regarding the recommended TOE before switching to oral treatment. In our view, the new class I recommendation for TOE should rather be considered a class IIa. There should be flexibility to omit it in straightforward cases with optimal visualization on TTE, especially since, in the POET trial it did not alter treatment decisions for any patients [40].

### Surgical indication

**Fig. 5:**
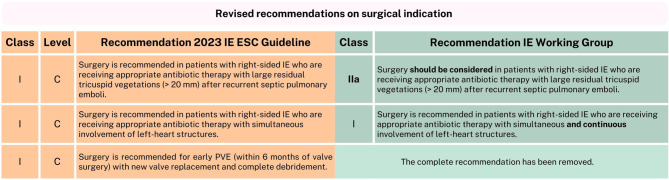
Revised recommendations on surgical indication. *IE* infective endocarditis, *ESC* European Society of Cardiology, *mm* millimeter, *PVE* prosthetic valve endocarditis

The three main categories that indicate surgery in IE patients remain heart failure, uncontrolled infection, and prevention of embolic events [1]. The IE Working Group has endorsed most of the new guidelines’ surgical recommendations. However, there are some remarks regarding one left-sided IE recommendation, two right-sided IE recommendations, and the new recommendation for early PVE (Fig. 5). In addition, the new guideline introduced a general change in the timing of performing surgery for IE. Urgent surgery is now classified as within 3–5 days, as well as leaving it to the discretion of the Endocarditis Team (while not delaying surgery by waiting on the Endocarditis Team) [1]. We endorse this change in surgical timing, as the trend in most studies has been to perform surgery quickly in order to reduce mortality, most of all in patients with heart failure or prevention of an embolism as the surgical indication [51–55].

Another general change since the 2015 guideline is the adoption of a different classification for vegetation length. The previous guideline made a distinction between vegetations > 10 millimeters (mm), > 15 mm and > 30 mm in their surgical recommendations, while the 2023 guideline only distinguishes between vegetations > 10 mm and those smaller for formal surgical recommendations [1, 2]. Although most (limited) studies seem to suggest that early surgery may benefit patients who are at higher risk of embolization, the available data on this, including specific vegetation sizes, remain insufficient [51, 53, 54, 56, 57]. Individualized decision-making is therefore recommended for this (now to a greater degree) heterogeneous group. In line with this, the new IIb recommendation for urgent surgery in left-sided IE with a vegetation ≥ 10 mm, without severe valve dysfunction or without clinical evidence of embolism and low surgical risk, was evaluated. The IE Working Group endorses this recommendation because it addresses this heterogeneous group, and class IIb allows for the flexibility needed. For this patient group, it remains unclear whether surgery or conservative treatment is more effective. The ongoing randomized controlled ASTERIx trial may provide clarity in the future [58]. As for the new right-sided IE surgical indications, the IE Working Group could endorse most of them. However, two specific recommendations warrant further consideration. The first is the new class I recommendation for surgery in patients with right-sided IE who are receiving appropriate antibiotic therapy with large residual tricuspid vegetations (> 20 mm) after recurrent septic pulmonary emboli. We suggest allowing some flexibility (which a class I indication does not), as none of the presented evidence supports this recommendation [56, 59]. A IIa indication would allow deviation, for example, in cases where patients lack relevant tricuspid valve insufficiency and because emboli are generally less alarming from a right-sided source [60].

The second new class I indicated for surgery in patients with right-sided IE to review is that of patients with simultaneous involvement of left-heart structures. The IE Working Group specifies this recommendation to ‘simultaneous and continuous involvement’ [1]. The IE Working Group does not consider a class I indication appropriate for patients with solely left- and right-sided endocarditis (without continuous involvement and) without other surgical indication(s).

Finally, a new class I, level C recommendation was made by the new guideline, which recommends surgery for early PVE (within 6 months of valve surgery) with new valve replacement and complete debridement [1]. The first year after implantation is considered a vulnerable period due to increased healthcare contact and still ongoing endothelialisation of the prosthetic valve. Moreover, the in-hospital mortality rate seems to be higher in the early PVE group as opposed to patients with PVE later after implantation [61, 62]. Given this knowledge, we consider the new focus on this high-risk group (early PVE) in the guideline appropriate. However, there is no supporting evidence for the specific recommendation itself, much less a class I indication.

## Neurological complications and surgical indication

The new guideline introduces changes regarding the advised timing of cardiac surgery following neurological complications, with a stronger emphasis on surgical treatment options than previously [1, 2]. All recommendations were endorsed by the IE Working Group, although there are some optimizations proposed for the clinical (neurological) criteria to consider prior to surgical decision-making (Tab. 3).

**Table 3:** Revised clinical (neurological) criteria in case of cardiac surgery following neurological complications of IE.

Features of:	Description of IE ESC guideline	Description of IE Working Group
Severely decreased level of consciousness	Glasgow Coma Scale ≤ 4 or NIHSS > 18	Glasgow Coma Scale *sum score* ≤ 4 *due to structural imaging proven brain lesions *or National Institutes of Health Stroke Scale Score (NIHSS) > 18
Favourable brain bleed	Intracranial haemorrhage volume of < 30 mL or NIHSS < 12	Intracranial *supratentorial* haemorrhage volume < 30 mL or NIHSS *<* *15*

*IE* infective endocarditis, *ESC* European Society of Cardiology, *NIHSS* National Institutes of Health Stroke Scale Score

The most important changes compared to the former guideline encompass the following. First, the upgraded (from IIa to) class I, level B recommendation ‘to perform surgery without any delay after stroke (in the presence of heart failure, uncontrolled infection, abscess formation, or persistent high embolic risk, as long as coma is absent, and the presence of cerebral haemorrhage has been excluded by cranial CT or MRI)’. Second, for intracranial haemorrhage, the addition to consider frequent re-assessment was added to the known recommendation to delay cardiac surgery for > 1 month. Finally, a completely new IIa, level C recommendation was introduced as well, ‘to consider urgent or emergency surgery in patients with intracranial haemorrhage and unstable clinical status (due to heart failure, uncontrolled infection or persistent high embolic risk), all the while weighing the likelihood of a meaningful neurological outcome’.

Regarding our proposed adjustments for the clinical neurological criteria before a potential IE surgery, the ESC guideline describes a severely decreased level of consciousness as ‘a Glasgow Coma Scale ≤ 4 or National Institutes of Health Stroke Scale Score (NIHSS) > 18’. We would specify the first criterion to ‘Glasgow Coma Scale sum score ≤ 4 due to structural imaging proven brain lesions’, to clarify and exclude other possible causes of the comatose condition. Furthermore, favourable brain bleed features are described as ‘an intracranial haemorrhage volume of < 30 mL or NIHSS < 12’, which we believe should be intracranial supratentorial haemorrhage volume < 30 mL or NIHSS < 15. The addition of supratentorial, given that this volume of bleeding would be fatal in the brainstem or cerebellum, and NIHSS < 15 (instead of < 12), because NIHSS scores of 5‑15 indicate moderate strokes with favourable outcomes, and we find such cases all deserve to be discussed for an indicated surgery in the Endocarditis Team (Tab. 3). In conclusion, there is currently insufficient evidence to support modification of the cutoff value widely used in clinical practice [63–65].

## Cardiac implantable electronic devices

**Fig. 6:**
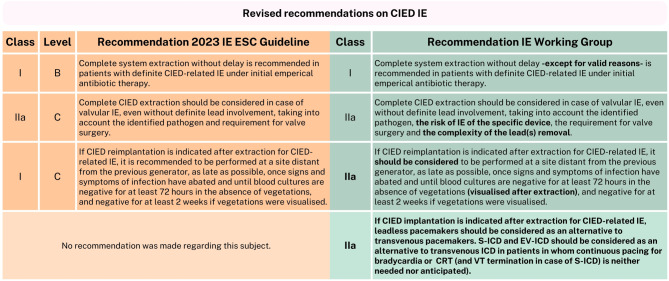
Revised recommendations on CIED IE. *IE* infective endocarditis, *ESC* European Society of Cardiology, *CIED* cardiac implantable electronic device, *S‑ICD* subcutaneous implantable cardioverter-defibrillator, *EV-ICD* extravascular implantable cardioverter defibrillator, *ICD* implantable cardioverter defibrillator, *VT* ventricular tachycardia, *CRT* cardiac resynchronization therapy

The IE Working Group endorses nearly all CIED-related recommendations; however, there are some considerations and comments, and one new proposed recommendation (Fig. 6).

The new class IIa, level C recommendation, that ‘immediate epicardial pacemaker implantation should be considered in patients undergoing surgery for valvular IE and complete atrioventricular block if one of the established predictors is present’ is endorsed, as the opportunity naturally presents itself during thoracotomy and the procedure is straightforward, safe and potentially reduces the delay to another pacemaker implantation [1, 66]. In addition, the class IIa indication leaves flexibility to adjust this treatment approach if it’s not desirable in an individual patient case. Moreover, it is worth noting that a leadless pacemaker can also be implanted during surgery as well as peri-operative in collaboration with an experienced electrophysiologist [67–69]. As for the new class I, level B indication that ‘complete system extraction is recommended without delay in patients with definite CIED-related IE’, there are some aspects to reflect on. The trend towards earlier extraction is understandable as there is no rationale to wait, like with PVE (the theory to treat with antibiotics for some time before intervention) [70, 71]. However, ‘without delay’ should not necessarily be perceived as an urgent indication. In some cases, there may be reasons to wait, as percutaneous extraction is usually preferred over surgical, however large vegetations or hemodynamic instability may make percutaneous extraction impossible at the initial stage. Lastly, although this is a class I recommendation, in practice this specific recommendation will require more careful consideration and shared decision-making, as frailty, comorbidities, and high extraction risk are common in this population, and there are cases where conservative treatment is effective [72].

In the area of CIED extraction, the 2015 class IIb, level C recommendation that ‘complete CIED extraction may be considered in case of valvular IE and an intracardiac device with no evidence of device infection’, was upgraded to class IIa, level C and nuanced by the addition ‘to take into account the identified pathogen and requirement for valve surgery’ [1, 2]. We propose to further extend the recommendation to also take into account the complexity of the lead(s) removal, considering factors such as the age of the leads and the presence of abandoned leads, as well as the risk of IE of the specific device, for example, weighing the lower risk of IE for leadless pacemakers [73–77]. The 2015 class IIa, level C indication for complete hardware removal on the basis of occult bacteraemia without another apparent source of infection, was refined with the addition of ‘in case of persistent/relapsing bacteraemia after a course of antimicrobial therapy’ and the recommendation was split into a IIa, level C recommendation for occult Gram-positive bacteraemia or fungaemia and a class IIb, level C, recommendation for occult Gram-negative bacteraemia [1, 2]. We endorse these changes, stressing the need to consider the severity of the causative pathogen and the associated risk of IE [78–81].

Another revised and upgraded in class (IIa to I) recommendation was issued, regarding reimplantation and its timing. The recommendation states: ‘if CIED reimplantation is indicated after extraction for CIED-related IE, it is recommended to be performed at a site distant from the previous generator, as late as possible, once signs and symptoms of infection have abated and until blood cultures are negative for at least 72 h in the absence of vegetations, and negative for at least 2 weeks if vegetations were visualised’. There is still insufficient evidence for the best timing of reimplantation after CIED IE [82]. This new recommendation provides more direction for the timing of reimplantation than before, which in practice, is appreciated for the treating physician. However, we would change the recommendation to class IIa, factoring in the lack of supporting evidence for the reported timing [83, 84]. Additionally, we would clarify the term ‘vegetations’, as they specifically imply vegetations found after extraction (also called ‘ghosts’), which have been associated with mortality and reinfection [85].

Finally, the new guideline remains conservative in its recommendations regarding device choice for reimplantation following CIED-related IE. The options for alternative devices, such as leadless pacemakers and subcutaneous implantable cardioverter defibrillators (S-ICD’s), are mentioned, but no formal recommendations were made regarding them. In addition, the extravascular implantable cardioverter defibrillator (EV-ICD) is also a valid option for many implantable cardioverter defibrillator carriers [86]. We advocate that these options should be considered as alternatives to transvenous systems, citing increased clinical experience, reduced infection risk demonstrated by results from various studies, such as the Micra postapproval registry, along with support from other established and widely used guidelines [87–92]. The ESC guideline on cardiac pacing and cardiac resynchronization therapy recommends (class IIa, level B) implanting leadless pacemakers in patients with previous infection and the American guideline for management of patients with ventricular arrhythmias and the prevention of sudden cardiac death includes the (class I) recommendation for S‑ICD implantation in patients at high risk of infection. It is important to note that appropriate patient selection remains key, taking into account all patient-specific circumstances [87, 91, 93].

## Conclusion

The multidisciplinary IE Working Group reviewed all 2023 ESC recommendations on the management of IE and concluded that most could be endorsed. However, specific modifications are recommended to more accurately reflect the strength of the underlying evidence and to better align with the national context. Key adjustments include a simplified antibiotic prophylaxis regimen, revised diagnostic, surgical and device recommendations and an adjusted recommendation for oral antibiotic therapy, in line with the anticipated revised Dutch Working Group on Antibiotic Policy (SWAB) IE guideline. This review provides guidance for IE management within the Dutch clinical setting.

## Supplementary Information

*Patient education*
A physical version of the patient information card will be distributed nationally as widely as feasible. Conveniently, the inclusion of the QR code enables immediate and easy digital storage and sharing, ensuring broad accessibility. For English-speaking patients, we refer to the patient education card provided in the 2023 ESC guidelines on the management of infective endocarditis.
Fig S1. Dutch patient information card to prevent infective endocarditis in patients at high risk.
